# Benefits of Huang Lian mediated by gut microbiota on HFD/STZ-induced type 2 diabetes mellitus in mice

**DOI:** 10.3389/fendo.2023.1120221

**Published:** 2023-01-18

**Authors:** Dan Li, Guangli Feng, Yue Li, Han Pan, Pei Luo, Bo Liu, Tao Ding, Xin Wang, Huibo Xu, Yufeng Zhao, Chenhong Zhang

**Affiliations:** ^1^ State Key Laboratory of Microbial Metabolism, School of Life Sciences and Biotechnology, Shanghai Jiao Tong University, Shanghai, China; ^2^ Pharmacodynamics and Toxicology Evaluation Center, Jilin Provincial Academy of Traditional Chinese Medicine, Jilin, China

**Keywords:** Huang Lian, type 2 diabetes mellitus, gut microbiota, bile acids, microbial BA metabolism

## Abstract

**Background:**

Huang Lian (HL), one of the traditional Chinese medicines (TCMs) that contains multiple active components including berberine (BBR), has been used to treat symptoms associated with diabetes for thousands of years. Compared to the monomer of BBR, HL exerts a better glucose-lowering activity and plays different roles in regulating gut microbiota. However, it remains unclear what role the gut microbiota plays in the anti-diabetic activity of HL.

**Methods:**

In this study, a type 2 diabetes mellitus (T2DM) mouse model was induced with a six-week high-fat diet (HFD) and a one-time injection of streptozotocin (STZ, 75 mg/kg). One group of these mice was administrated HL (50 mg/kg) through oral gavage two weeks after HFD feeding commenced and continued for four weeks; the other mice were given distilled water as disease control. Comprehensive analyses of physiological indices related to glycolipid metabolism, gut microbiota, untargeted metabolome, and hepatic genes expression, function prediction by PICRUSt2 were performed to identify potential mechanism.

**Results:**

We found that HL, in addition to decreasing body fat accumulation, effectively improved insulin resistance by stimulating the hepatic insulin-mediated signaling pathway. In comparison with the control group, HL treatment constructed a distinct gut microbiota and bile acid (BA) profile. The HL-treated microbiota was dominated by bacteria belonging to Bacteroides and the Clostridium innocuum group, which were associated with BA metabolism. Based on the correlation analysis, the altered BAs were closely correlated with the improvement of T2DM-related markers.

**Conclusion:**

These results indicated that the anti-diabetic activity of HL was achieved, at least partly, by regulating the structure of the gut microbiota and the composition of BAs.

## Introduction

Type 2 diabetes mellitus (T2DM) was the predominant form of diabetes in about 426 million people worldwide in 2017, thereby becoming a major public health problem (1). The prevalence of T2DM may be related to multiple risk factors, such as genetics, and environmental factors (e.g., eating pattern, physical inactivity) (2, 3). Because of the complex pathogenesis, the underlying mechanism of T2DM is still inconclusive. Emerging evidence shows that gut microbiota is an important factor in the development of T2DM (4). Several mechanisms of gut microbiota acting on the glycolipid metabolism of the host have been revealed, for example, bile acids (BAs) that are modified by the gut microbiota, serving as signaling molecules to target farnesoid X receptor (FXR) and G protein-coupled bile acid receptor-1 (TGR5), were found to promote the secretion of glucagon-like peptide-1 (GLP-1) to maintain glucose homeostasis (5), induce thermogenic genes and inhibit lipogenic genes to inhibit obesity (6). Notably, the metabolic benefits of some oral antidiabetic medications were found to be partly mediated by gut microbiota (7–10). For example, metformin, the first-line drug of T2DM, effectively improved glucose homeostasis in T2DM patients partially by regulating the *Bacteroides fragilis*-glycoursodeoxycholic acid (GUDCA)-intestinal FXR axis (11).

In China, Traditional Chinese Medicine (TCM) also serves as an important therapy for managing T2DM, and the efficacy of TCM might be closely related to the gut microbiota. Previously, several mechanisms of TCM in glycolipid metabolism have been reported *in vitro*, such as the up-regulation of the hepatic low-density lipoprotein receptor (12), promotion of intestinal GLP-1 secretion (13), and stimulation of AMPK activity in both myotubes and adipocytes (14). Due to the low bioavailability after oral administration, the maximum concentration (C_max_) of TCM in plasma is too low to reach the effective concentration for targeted regulation *in vitro* studies (15, 16). Therefore, the efficacy of TCM cannot be elucidated solely from the perspective of oral absorption, which might be mediated by gut microbiota. The active ingredients in TCM were shown to be able to regulate the structure and function of the gut microbiota. TCM, however, can be metabolized by gut microbiota to allow easy absorption (17, 18). Berberine (BBR), a monomer from TCM, is used as an effective drug for managing T2DM, which was found to regulate the gut microbiota (19–21). Growing evidence has reported that the anti-diabetic effect might be involved in the modulation of microbial short-chain fatty acids and BA metabolism by BBR (19, 22). A study reported that BBR could effectively alleviate the level of hemoglobin A1c in T2DM patients, which was associated with the inhibition of *Ruminococcus bromii* and its deoxycholic acid (DCA) conversion (23). Thus, investigations on the participation of the gut microbiota in T2DM may reveal new insights for a better understanding of the mechanisms of TCM in treating diabetes.

Compared to BBR, Huang Lian (HL, known as *Coptidis Rhizoma*) which is a TCM containing BBR, exerts a better glucose-lowering effect in mice and shows lower cytotoxicity in HepG2 cells (24–26). This might reflect that multiple ingredients within HL could regulate different pathways/targets to enhance pharmacological potency of HL. Previously, a study found that the modulation effects of HL and BBR on the gut microbiota were different, suggesting that other ingredients, except BBR, also played a significant role in modulating the gut microbiota (27). However, the role of the altered gut microbiota by HL in treating diabetes is still unclear.

In this study, a T2DM mouse model induced by HFD/STZ and an ethanol extraction of HL (28) were used to explore the mechanisms underlying the effect of HL on T2DM. The HL extract contains a total of 69.4% alkaloids. We found that HL treatment markedly improved insulin sensitivity and blunted fat accumulation in mice, which were closely associated with the modulation of microbial BA metabolism and the increase in BA synthesis. Our findings provide fundamental knowledge regarding the benefits of HL for T2DM and suggest that the gut microbiota is a potential target for HL to mitigate T2DM.

## Material and methods

### Extraction of HL extract

The herbal of Huang Lian (origin: Coptis chinensis Franch,; source: Mianyang, Sichuan) was purchased from Anhui Wugeng Traditional Chinese Medicine Pieces Co., Ltd. (Bozhou, China) and identified by Jilin Provincial Academy of Traditional Chinese Medicine according to the Chinese Pharmacopeia method. HL was stored in a ventilated and dry place. And HL extract was prepared as follows. The small pieces of HL were refluxed with 5-fold of 70% ethanol (1:5, w/v) twice for 2 h. Two batches of filtrates were combined, and pH value was adjusted to 1.5-2.0 with hydrochloric acid. The mixture was subsequently dissolved using NaCl. After being refrigerated for 48 h, precipitation was dried by vacuum concentration to obtain HL extract. Then extract was grinded into powder and stored in seal at 4°C. The freeze-dried powder was supplied by Jilin Provincial Academy of Traditional Chinses Medicine and was dissolved in distill water when used in the experiment.

### Quantitative analysis of marker components of HL extract

The contents of marker components within HL extract were analyzed with high performance liquid chromatography (HPLC) method. 25 mL solution (methanol: hydrochlori acid = 100: 1) was added to 40 mg extract powder and the mixture was ultrasound for 20 min. Then mixture was diluted 10 times with solution (methanol: hydrochlori acid = 100: 1) and well vortexed. After centrifugation, the supernatant was analyzed using HPLC system to evaluate the content of epiberberine, coptidis, palmatine, and berberine. HPLC was performed on LC-Q10 AT system, consisting of a binary solvent delivery manager, an auto-sampler, and a UDV detector. Chromatographic separations were performed on an Agilent C18 column (4.6 × 250 mm,5 μm). Flow rate and column temperature were set at 1 ml/min and 25°C, respectively. The injection volume was 10 µL, and the solvent was filtered through 0.45 µm Millipore filter and degassed prior to use. Briefly, acetonitrile (A) and 0.05% potassium dihydrogen phosphate mixed with 0.4% sodium dodecyl sulfate (pH was regulated to 4.0 by phosphoric acid) (B) (50:50) were used as mobile phase. Ultraviolet detection with the wavelength at 345 nm and was used for the determination of epiberberine, coptidis, palmatine, and berberine.

### Animal experiments

Male C57BL/6 mice (5 weeks old) were purchased from Slake experimental Animal Co., Ltd. (Shanghai, China). All mice were housed in a temperature-controlled room (22 ± 3°C) under specific pathogen-free (SPF) conditions with a standard 12 h light/dark cycle. All animal experimental procedures were approved by the Institutional Animal Care and Use Committee (IACUC) of Shanghai Jiao Tong University (No. A2020030). Because there is a certain risk of death in STZ injection, we used additional three and two mice to DM group and DM-HL group, respectively. When we performed the randomly grouping of the mice, the mice were weighted and sorted in order and divided into multiple groups according to body weight. During the experiment, none of mice died with STZ or HL treatment. Eventually, mice were divided into the NC group (n = 8), DM group (n = 11), and DM-HL group (n = 10). The mice in the NC group were fed a normal chow diet (NC, 12450J, 10% calories from fat, 70% calories from carbohydrate, 20% calories from protein, 3.85 kcal/g; produced by Fanbo Biotechnology Co., Ltd., Shanghai, China); other mice were fed a high-fat diet (HFD, D12492, 60% calories from fat, 20% calories from carbohydrate, 20% calories from protein, 5.24 kcal/g; produced by Fanbo Biotechnology Co., Ltd., Shanghai, China). Two weeks after dietary intervention commenced, the mice in the DM-HL group were orally gavaged daily with a dosage of 50 mg/kg HL extract, which continued until the end of the experiment. The mice in the NC and DM groups were gavaged daily with an equivalent volume of distilled water as a control. After the 4-week diet intervention commenced, the mice in the DM and DM-HL groups were injected intraperitoneally with a single dose of streptozotocin (STZ, 75 mg/kg) (S0130, Sigma-Aldrich, Germany) to construct the T2DM model. At the same time, the mice in the NC group received the equivalent volume of citric buffer to eliminate the effects of the injection procedure. All mice were allowed ad libitum access to water and food during the trial. The trial lasted for 6 weeks in total. Body weight was monitored weekly. Fecal samples were collected in week 6 and stored at −80°C for gut microbiota analysis.

All mice were fasted for 6 h before sampling. Blood samples were collected from the orbital vascular plexus. Serum samples were isolated from blood samples after centrifugation for 15 min at 4°C at 3000 g and then stored at −80°C. Liver, adipose tissues (epididymal, retroperitoneal, perirenal), and colon contents were weighed and collected immediately and stored at −80°C or in 4% paraformaldehyde for further analyses.

### Oral glucose tolerance test (OGTT)

After 6 h of fasting, all mice were administered a glucose solution (2 g·kg^-1^ body weight) through oral gavage. The glucose levels at 0 min (which is fasting glucose) and 15, 30, 60, 90, and 120 min after the glucose challenge were measured using a blood glucometer (ACCU-CHEK Performa, Roche, USA). Blood samples at 0 min, 15 min, and 60 min after the glucose challenge were collected from the tip of the tail vein. Serum samples were isolated for insulin detection after centrifugation at 4°C at 3000 rpm for 15 min. The insulin concentration was measured using an ultrasensitive mouse insulin ELISA kit (90080, Crystal Chem, USA).

### Histological analysis of epididymal fat and liver

Fresh epididymal fat and liver were fixed with 4% paraformaldehyde and embedded in paraffin. Then, 4-μm sections were stained with hematoxylin and eosin (H&E) (G1003, Wuhan Servicebio Technology Ltd., China). The adipocyte area and nonalcoholic fatty liver disease (NAFLD) activity score were analyzed using Image Pro Plus v6.0 software (Media Cybernetics Inc., Silver Springs, USA). For epididymal fat, at least 300 adipocytes of each mouse were required to assess the mean area of adipocytes under ×100 magnification. For the liver, three discontinuous scans of each mouse were used to assess the NAFLD activity score under ×100 magnification as previously described (29).

Epididymal fats were fixed overnight with 4% paraformaldehyde. In accordance with the standard process, the tissue was sectioned and stained with Oil Red O. The slices were imaged under microscope.

### Analysis of protein expression by Western Blot

Frozen livers were homogenized using a tissue homogenizer in pro-cooled 1× RIPA buffer (ab156034, Abcam, Cambridge), containing protease and phosphatase inhibitors (P1045, Beyotime, China) (30). Then, homogenates were lysed for 5 min at 25 Hz/s using a TissueLyser (Qiagen, Germany). Protein was isolated from homogenates after centrifugation at 4°C at 12000 rpm for 10 min. A BCA protein Assay Kit (P001P, Beyotime, China) was used to quantify the protein concentration according to the manufacturer’s instructions. The protein was boiled at 100°C for 10 min after an equal volume of 2× SDS loading buffer (P0015B, Beyotime, China) was added. An equal amount of protein across all samples was separated on 10% SDS-PAGE gels (180-9117HA, Tanon, China) and diverted immediately to PVDF membranes (G6044-0.45, Servicebio, China). After 2 h of blocking in 8% skimmed milk at room temperature, the membranes were incubated with the primary antibodies overnight at 4°C. After washing 3 times with 0.1% TBST solution, the membranes were incubated with secondary antibodies for 2 h at room temperature. Eventually, the bands were visualized using the ECL stain kit (180-501, Tanon, China), and the intensity of the bands was determined by Image Pro Plus v6.0 software. Primary antibodies were as follows: IRS1 (1:2000, ab131487, Abcam), p-IRS1 (1:3000, 2386, cell signaling), Akt (1:2000, 9272, cell signaling), p-Akt (1:1000, 9271, cell signaling), Glut4 (1:2000, ab33780, Abcam), and GAPDH (1:3000, ab9485, Abcam).

### Quantification of mRNA expression by RT-qPCR

Total RNA was extracted from liver tissues using a RNeasy minikit (74804, Qiagen, Germany). Then, reverse transcription was performed to synthesize cDNA using a SuperScript kit (18080-051, Invitrogen, USA). According to the manufacturer’s instructions, cDNA was then amplified using real-time PCR (qPCR) to determine the expression of *Cyp27a1*, *Cyp7b1*, *Cyp7a1*, and *Cyp8b1* in a 20-μL reaction system containing cDNA template, forward primer, reverse primer, ddH_2_O, and SYBR Green I PCR Supermix (Bio-Rad). The reaction conditions of forty cycles were as follows: 95°C for 20 s, 56°C for 30 s, and 72°C for 30 s, followed by plate reads for 5 s. Gene expression levels were calculated using the ΔΔ*C_T_* method and normalized to *β-actin*. The forward and reverse primer sequences of target genes and *β-actin* were listed as follows (31, 32). *Cyp27a1* gene, F: 5'-CCAGGCACAGGAGAGTACG-3'; R: 5'-GGGCAAGTGCAGCACATAG-3'. *Cyp7b1* gene, F: 5'-GGAGCCACGACCCTAGATG-3'; R: 5'-TGCCAAGATAAGGAAGCCAAC-3'. *Cyp7a1* gene, F: 5'-AGCTCTGGAGGGAATGCCAT-3'; R: 5'-GAGCCGCAGAGCCTCCTT-3'. *Cyp8b1* gene, F: 5'-CCTCTGGACAAGGGTTTTGTG-3'; R: 5'-GCCATCAAGGACGTCAGCA-3'. *β-actin* gene, F: 5'**-**GGCTGTATTCCCCTCCATCG-3'; R: 5'-CCAGTTGGTAACAATGCCATGT-3'.

### Isolation of fecal DNA

Total DNA was extracted from week 6 fecal samples according to a procedure published previously (33). DNA concentration was determined by a NanoPhotometer and expressed in micrograms (μg). The DNA concentration across all samples was diluted to 10 ng/µL for subsequent analysis.

### Total bacterial loads assessed by qPCR

A plasmid containing the 16S full-length *Roseburia inulinivorans* strain was diluted successively to 10^9^,10^8^, 10^7^, 10^6^, 10^5^, 10^4^, 10^3^, and 10^2^ copies/μL. According to the manufacturer’s instructions, qPCR was performed in a 20-µL reaction system containing a DNA template, Uni331F primer (5'-TCCTACGGGAGGCAGCAGT-3'), Uni797R primer (5'-GGACTACCAGGGTATCTAATCCTGTT-3') (34), ddH_2_O, and SYBR Green I PCR Supermix (Bio-Rad). The reaction conditions of forty cycles were as follows: 95°C for 10 s, 65°C for 60 s, and 80°C for 5 s, followed by plate reads for 5 s. A standard curve was constructed according to the linearity between the copy number of plasmids and the *C_T_* value. The copy number of DNA samples was calculated according to the standard curve.

### Analysis of 16S rRNA gene V3-V4 sequencing

An amplicon library was prepared by amplifying the V3-V4 region of the 16S rRNA gene according to previously published procedures (35). To profile the composition of microbial communities, high-throughput sequencing of the prepared amplicon library was performed on an Illumina Miseq platform (Illumina, Inc., USA).

Raw paired-end reads were analyzed with Quantitative Insights into Microbial Ecology2 software (QIIME2, v2021.4). After the removal of the adapters and primers with the “Cutadapt” plugin, DADA2 was used to obtain amplicon sequence variants (ASVs) through denoising, merging, and filtering. Then, a rooted phylogenetic tree was constructed with the “FastTree” plugin based on representative sequences of ASVs. Subsequently, the taxonomy of ASVs was identified based on the Silva138 16S rRNA database. In combination with the quantified total bacterial load, the relative abundance of ASVs was converted into absolute abundance for subsequent analysis (36, 37).

Redundancy analysis (RDA) was performed using Canoco 5 software (Microcomputer Power, USA) to identify key variations of ASVs responding to the T2DM model and HL treatment, which drive the segregation of the gut microbiota among groups. A heatmap representing the abundance variations of the key ASVs generated from the RDA model was constructed using the “Complexheatmap” R package. Pairwise comparisons (DM *vs.* NC, DM-HL *vs.* DM, DM-HL *vs.* NC) of ASVs were assessed using a Mann-Whitney U test. Microbial functional genes were predicted using PICRUSt2 based on the Greengenes database of 16S rRNA sequences (38) and the Kyoto Encyclopaedia of Genes and Genomes (KEGG) database.

### Untargeted metabolomics analysis of the colon content

The colon content was homogenized in 600 µL 2-chlorophenylalanine (4 ppm) methanol and vortexed for 30 s. Then, homogenates were sonicated at room temperature for 10 min. The supernatants were collected after centrifugation (4°C, 12000 rpm, 10 min) and filtration with a 0.22-µm membrane for high-performance liquid chromatography-mass spectrometry analysis (HPLC-MS/MS). Quality control (QC) samples were prepared with the same procedures. HPLC-MS/MS was performed by Suzhou PANOMIX Biomedical Tech Co., Ltd. (Suzhou, China). Chromatographic separation was performed with an ACQUITY UPLC® HSS T3 (150 × 2.1 mm, 1.8 μm, water) column maintained at 40°C. Data-dependent acquisition in the MS/MS experiment was operated with an HCD scan. The raw data of metabolites were converted into mzXML format with Proteowizard software. Then, perk identification, filtration, and alignment were performed using the “XCMS” R package. The output metabolites were identified based on the Metlin metabolites database (metlin.scripps.edu), MoNA metabolite database (mona.fiehnlab.ucdavis.edu), and in-house MS2 database.

Variable importance in projection (VIP) scores calculated by the orthogonal projection to latent structure-discriminant analysis (OPLS-DA) model were used to estimate the importance of each variable to drive the segregation of the metabolome among the two groups. Through pairwise comparison between groups (DM *vs.* NC, DM-HL *vs.* DM, DM-HL *vs.* NC), differential metabolites were selected based on the following criteria: 1) VIP > 1.5 in the OPLS-DA model, 2) fold-change > 2, and 3) *p* < 0.05 (including methods used for testing and *p* value correction). MetaboAnalyst was used for the analysis of pathway enrichment (MetaboAnalyst).

### Statistical analysis

Statistical analysis was performed using GraphPad Prism version 8.0 (GraphPad Software, Inc., USA). Gut microbiota data were exhibited by a box plot, and significance was analyzed using one-way analysis of variance (ANOVA) followed by Tukey’s multiple comparisons. Physiological data, metabolites, and functional genes were expressed as the mean ± SEM, and significant differences were evaluated by one-way ANOVA or an unpaired *t* test with Welch’s correction. Spearman correlations were constructed with R software (v4.0.5). *P* < 0.05 is considered to be statically significant.

## Results

### HPLC analysis of HL extract

The chromatographic peaks of the main chemical constituents within HL extract were shown in **Supplementary Figure S1**. The contents of epiberberine, coptidis, palmatine, and berberine were determined to be 1.18%, 6.71%, 5.34%, and 44.34%, respectively. In short, the content of total alkaloids within HL extract was determined to be 69.43% using spectrophotometry.

### HL treatment blunted insulin resistance and fat accumulation

**Figure 1:**
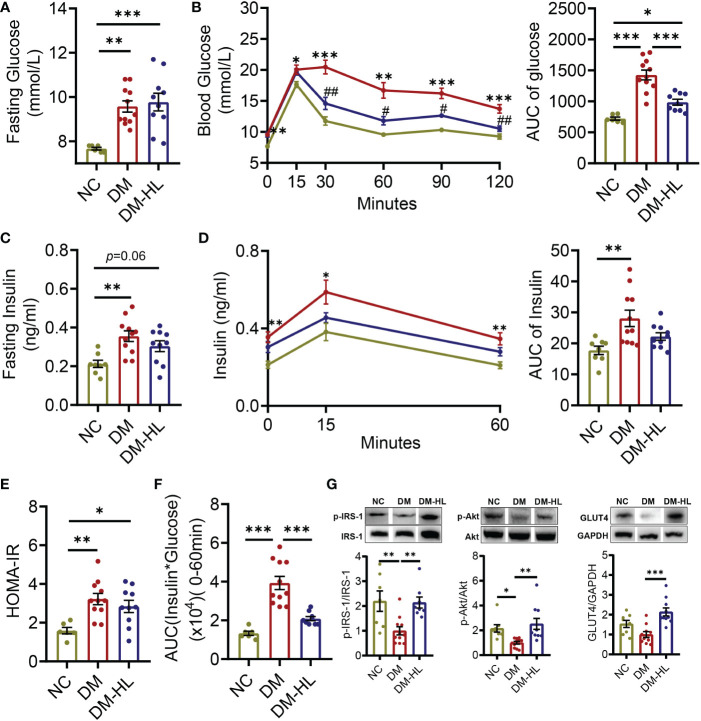
HL treatment improved glucose homeostasis and insulin sensitivity. **(A)** Fasting blood glucose. **(B)** Blood glucose levels and the AUC of blood glucose during OGTT. **(C)** Fasting serum insulin. **(D)** Serum insulin levels and the AUC of serum insulin during OGTT. **(E)** Homeostasis model assessment of insulin resistance (HOMA-IR). **(F)** Insulin resistance index (the product of the AUC of blood glucose multiplied by the AUC of serum insulin). **(G)** The phosphorylation of IRS1 and Akt, as well the expression of Glut4 in the liver. The relative abundances of p-IRS1, p-Akt, and Glut4 were normalized by IRS1, Akt, and GADPH, respectively. Data are presented as the mean ± SEM. Significance was evaluated using one-way ANOVA followed by Tukey’s *post-hoc* test. *p < 0.05, ** *p* < 0.01, ***p < 0.001. For the curve of graph **(B, D)**, *p < 0.05, **p < 0.01, ***p < 0.001 *vs.* NC group; # *p* < 0.05, ## *p* < 0.01 *vs.* DM group.

We used HFD with one streptozotocin (STZ, 75 mg/kg) injection to induce T2DM in C57BL/6 mice. One group of these mice was administered HL (50 mg/kg·day) by oral gavage after 2 weeks of feeding with HFD, which continued for an additional 4 weeks (DM-HL group), and the others were given distilled water as a disease control (DM group). At the end of the trial, fasting glucose and glucose levels during the oral glucose tolerance test (OGTT) were significantly higher in the DM group compared to that in the healthy mice (NC group) (**Figures 1A, B**). Fasting insulin, the area under the curve (AUC) of insulin during OGTT, HOMA-IR, and the insulin resistance index were also significantly higher in the DM group than in the NC group (**Figures 1C–F**). It was indicated that the mice in the DM group suffered glucose tolerance impairment and insulin resistance. Notably, although HL treatment had no effect on fasting glucose, fasting insulin, HOMA-IR, and insulin levels during OGTT, the mice in the DM-HL group had significantly reduced insulin resistance index and blood glucose levels (from the 30^th^ min during OGTT) compared to the DM group (**Figures 1A–F**), suggesting that HL treatment may increase insulin sensitivity but not insulin secretion. In the insulin signaling pathway, binding of insulin to the insulin receptor would induce the phosphorylation of insulin receptor substrates (p-IRS1), phosphorylate the downstream signaling substance Akt (p-Akt), and then increase the level of glucose transporter (Glut4), leading to glucose uptake (39). Thus, we measured the levels of p-IRS1, p-Akt, and Glut4 in the liver to evaluate the impact of HL on insulin sensitivity. We found that HL treatment reversed the down-regulation of the phosphorylation of IRS1 and Akt induced by HFD/STZ, and the level of Glut4 was dramatically higher in the DM-HL group than in the DM group (**Figure 1G**). These results suggested that HL intervention alleviated glucose tolerance by stimulating insulin sensitivity and glucose uptake.

**Figure 2:**
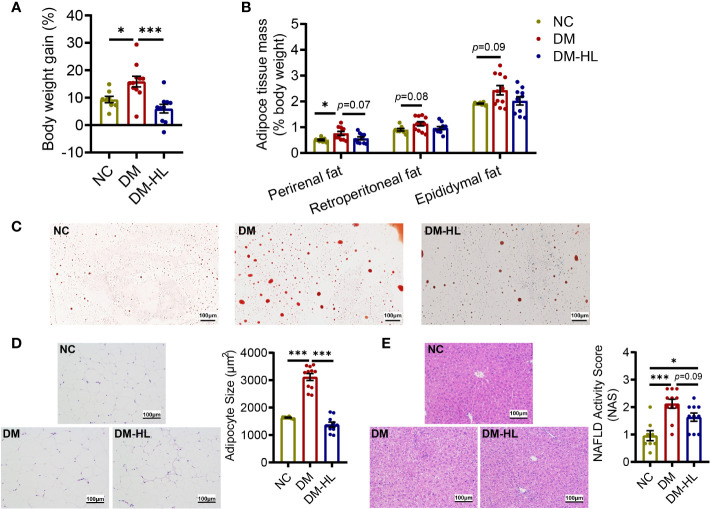
HL treatment blunted obesity and fat accumulation. **(A)** The body weight gain (% 1^st^ day body weight). **(B)** Perirenal, Retroperitoneal, and Epididymal mass (% body weight), respectively. **(C)** Red oil staining sections of epididymal fat (scale bar = 100 µm). **(D)** Representative H&E staining sections of epididymal fat (scale bar = 100 µm) and the mean adipocyte area. **(E)** Representative H&E staining sections of the liver (scale bar = 100 µm) and NAFLD activity score. Data are presented as the mean ± SEM. Significance was evaluated using one-way ANOVA followed by Tukey’s *post-hoc* test. *p < 0.05, ***p < 0.001.

In addition to impaired glucose tolerance, we also observed that the mice in the DM group had significantly higher body weight gain and over-accumulation of fat (**Figure 2**, **Supplementary Figure S2**). Across the experiment, HL treatment effectively inhibited excess body weight gain and successfully blunted lipid accumulation, as reflected by significantly lower body weight gain from week 3 during the experiment, as well as reduced adipocyte size and lipid droplets in epididymal fat and the NAFLD activity score in the DM-HL group than in the DM group (**Figure 2**, **Supplementary Figure S2**). These results suggest that HL treatment effectively inhibited obesity and fat accumulation induced by HFD/STZ.

### HL treatment shifted the structure of the gut microbiota

**Figure 3:**
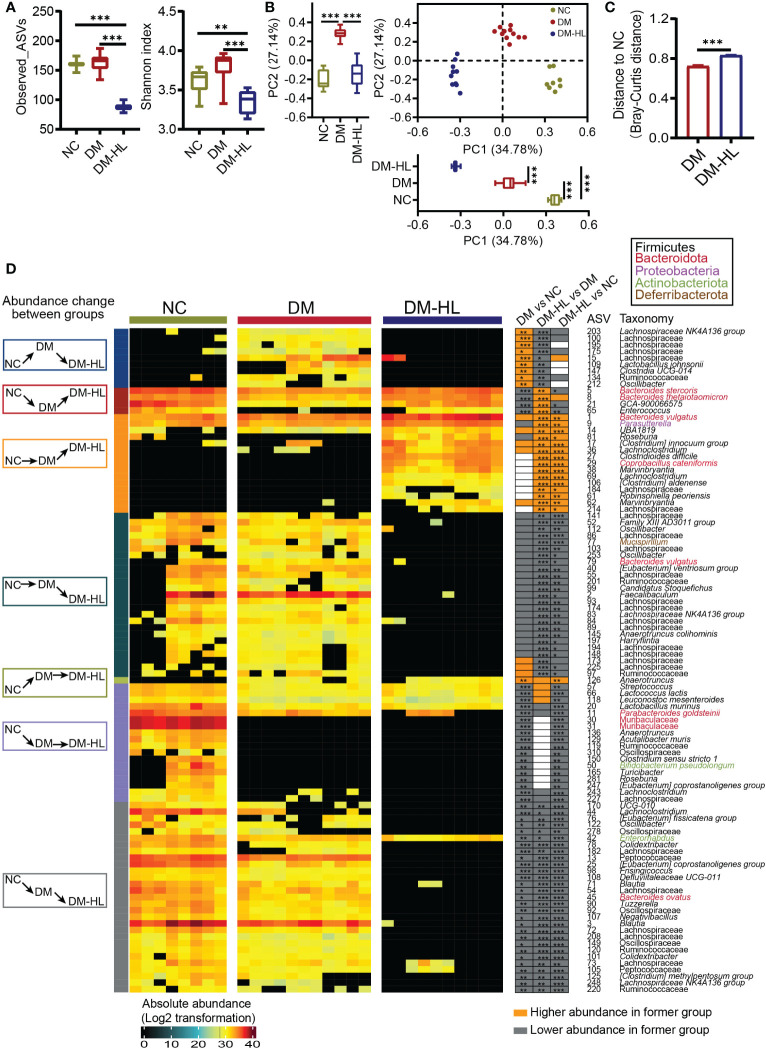
HL treatment significantly shaped a unique structure of the gut microbiota. **(A)** α-diversity of the gut microbiota represented as Observed_ASVs and the Shannon index. Data was shown in box plot. And the line in the middle of box is plotted at the median, the inferior and superior limits of the box correspond to the 25^th^ and 75^th^ percentiles, the whiskers correspond to maximum and minimum. Significance was analyzed using one-way ANOVA followed by Tukey’s *post-hoc* test. **(B)** Principal coordinate analysis (PCoA) of the gut microbiota based on the Bray-Curtis distance. The box plots showed the changes of gut microbiota among three groups on PC1 or PC2 (the line in the middle of box is plotted at the median, the inferior and superior limits of the box correspond to the 25^th^ and 75^th^ percentiles, the whiskers correspond to maximum and minimum). Significance was analyzed using one-way ANOVA followed by Tukey’s *post-hoc* test. **(C)** Bray-Curtis distance from the DM group and DM-HL group to the NC group. Data are presented as the mean ± SEM. Significance was analyzed using a two-tailed Mann-Whitney test. **p < 0.01, ***p < 0.001. **(D)** Heatmap of 101 ASVs across all samples. The color of the block corresponds to the log_2_-transformed value based on the absolute abundance of each sample. Left, several classifications of the change in ASV abundance among groups; frame colors indicate specific situations of change. Right, the changing direction of 101 ASVs through pairwise comparison of groups analyzed by the two-tailed Mann-Whitney test. Orange indicates that ASV abundance was higher in the latter group, and the grey indicates that ASV abundance was lower in the former group.

To determine the effects of HL treatment on the gut microbiota in T2DM mice, we collected fecal samples from all mice at the end of the trial for gut microbiota analyses. First, the total bacterial loads were determined by qPCR of the 16S rRNA gene. We found that the total bacterial loads of mice in the DM group and DM-HL group decreased by 3.8 times and 3.3 times, respectively, compared to the NC group, indicating significantly lower total bacterial loads in all HFD/STZ-treated mice (**Supplementary Figure S3**). Next, we sequenced the V3-V4 region of the 16S rRNA gene to analyze the structure and composition of the gut microbiota. Due to significant differences in the total bacterial loads among the three groups, the relative abundance of ASVs could not accurately reflect the alteration of the gut microbial structure (37). Therefore, for further analyses, we converted the relative abundance to the absolute abundance of ASVs based on the total bacterial loads of each sample. The richness and diversity of the gut microbiota, represented by the number of species and Shannon index, were similar between the DM group and NC group, while the mice in the DM-HL group had markedly reduced richness and diversity of gut microbiota, suggesting that HL may have antimicrobial activity against specific bacteria (**Figure 3A**). Principal coordinate analysis (PCoA) based on the Bray-Curtis distance at the ASV level showed obvious discriminations of the gut microbial structure among the three groups (**Figure 3B**). The structure of the gut microbiota in the DM group significantly separated from that in the NC group along PC1 (explained 34.78% of the total variance) and PC2 (explained 27.14% of the total variance). Specifically, compared to the DM group, the samples in the DM-HL group were more distinct from those in the NC group along PC1, while the samples in the DM-HL group were closer to those in the NC group along PC2. Notably, the Bray-Curtis distance of the gut microbiota in the mice between the DM-HL group and NC group was significantly higher than that between the DM group and NC group (**Figure 3C**). In short, HL did not reverse the gut microbiota disrupted by HFD/STZ treatment but constructed a strikingly distinct gut microbiota.

Redundancy analysis (RDA) based on the Monte Carlo permutation procedure (MCPP, *p* = 0.0001) also showed a significant effect of the T2DM model and HL treatment on the gut microbiota, which was consistent with the PCoA results. Then, we applied RDA to identify gut microbes responding to the T2DM model and/or HL treatment. Eventually, we selected 101 ASVs as key variations that accounted for at least 46.8% of the variability explained by both axes (**Supplementary Figure S4**). Compared to the NC group, the DM group significantly altered 61 ASVs (10 ASVs increased and 51 ASVs decreased). Among these 61 ASVs altered by the DM group, HL treatment changed 42 ASVs (4 ASVs increased and 38 ASVs decreased) and about one-third of them (13 out of 42 ASVs) were reversed to the same direction as the ASVs observed in the NC group. Of the 13 reversed ASVs, four were enriched (belonging to *Bacteroides*, *Enterococcus*, *Lachnospiraceae GCA-900066575*) and nine were reduced (belonging to Lachnospiraceae, Ruminococcaceae, *Lactobacillus*, *Clostridia UCG-014*, *Oscillibacter*) in the DM-HL group. The rest of the HL-changed ASVs (29 out of 42 ASVs) were significantly inhibited by HL treatment and most of them were eliminated in the DM-HL group except for ASV42 (belonging to *Enterornabdus*). In addition, the abundance of 40 ASVs belonging to various taxa were affected only by HL treatment, including the enrichment of 15 ASVs and the elimination of 25 ASVs in the DM-HL group. Overall, 76 out of 101 identified key ASVs were almost undetectable in the DM-HL group, including 34 ASVs belonging to the Lachnospiraceae family. Only 25 out of 101 identified key ASVs were predominant in the DM-HL group that accounted for about 48.6% of the total bacterial loads (12.1% in the NC group; 12.9% in the DM group), of which the top five abundant ASVs belonged to the *Bacteroides* (ASV1, ASV5, ASV8), *Parasutterella* (ASV9), and *Clostridium innocuum* (ASV17) groups. Moreover, we noticed that some ASVs in the same family or genus showed different behaviors. For example, among the 44 ASVs in the Lachnospiraceae family, 13 ASVs were increased while 31 ASVs were decreased in the DM-HL group compared with the DM group. Similar results were also found in the genera *Bacteroides* and *Roseburia*, indicating that the response of bacteria is species or even strain-specific (**Figure 3D**). These results showed a significantly distinct gut microbiota with lower total bacterial loads and diversity that developed in the DM-HL group.

### HL treatment changed the metabolites in the colon

**Figure 4:**
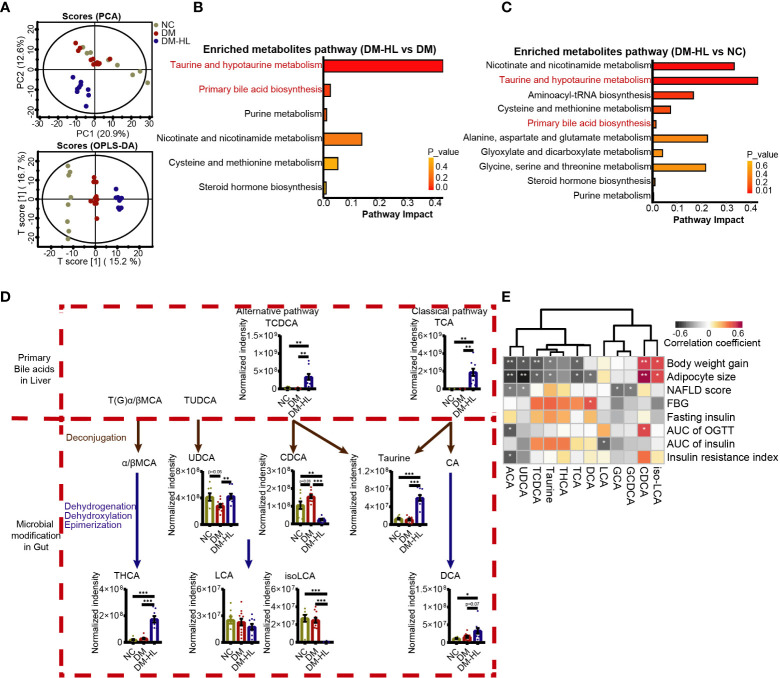
HL treatment altered the bile acid profile in the colon. **(A)** Principal component analysis (PCA) and orthogonal projection to latent structure-discriminant analysis (OPLS-DA) plot of the metabolome across all samples. **(B)** Enriched metabolite pathway in the DM-HL group compared to the DM group based on metabolic enrichment pathway analysis. **(C)** Enriched metabolite pathway in the DM-HL group compared to the NC group. **(D)** Metabolites involved in microbial bile acid metabolism in the gut. TCDCA, Taurochenodesoxycholic acid; TCA, Taurocholic acid; UDCA, Ursodeoxycholic acid; CDCA, Chenodeoxycholic acid; THCA, taurohyocholic acid; LCA, Lithocholic acid; isoLCA, isolithocholic acid; DCA, Deoxycholic acid. **(E)** Spearman’s correlation analysis between metabolites and T2DM-related parameters. For graph **(D)**, data are presented as the mean ± SEM. Significance was evaluated by an unpaired *t* test with Welch’s correction. *p < 0.05, **p < 0.01, ***p < 0.001.

To determine how the T2DM model and HL treatment impact the metabolites of the gut microbiota in mice, the untargeted metabolites of the colon of all mice were tested by HPLC-MS/MS. In total, we identified 541 metabolites under polar ionic mode. The principal-component analysis (PCA) and orthogonal projection to latent structure-discriminant analysis (OPLS-DA) exhibited obvious discriminations of metabolites among the three groups (**Figure 4A**, **Figure S5A**). Subsequently, we determined a total of 119 differential metabolites through pairwise comparisons between groups (VIP > 1.5 in the OPLS-DA model, fold-change > 2, and *p* < 0.05) (**Supplementary Figure S5B**; **Supplementary Table S1**). The number of differential metabolites in the mice between the DM-HL group and NC group was much higher than that between the DM group and NC group (71 vs. 37).

Among 119 differential metabolites, we recognized multiple herbal ingredients based on the TCMID database (http://www.megabionet.org/tcmid/), including BBR, tyrosol, yamogenin, 26-hydroxyecdysone, and 2,4-dihydroxyacetophenone, which may be present in the HL extract that was significantly enriched in the DM-HL group compared with the DM group (**Supplementary Figure S6**). Pathway enrichment analysis revealed that the differential metabolites between the DM and NC groups were mainly associated with nutrient and energy metabolisms, such as carbohydrate, lipid, amino acid, nucleotide, and cofactor and vitamin metabolisms. Notably, taurine and hypotaurine metabolism, and primary BA biosynthesis, which were highly related to microbial BA metabolism, were among the top 5 pathways enriched in the DM-HL group compared to the DM and NC groups (**Figures 4B, C**; **Supplementary Figure S7**). It has been reported that members of the gut microbiota can deconjugate the conjugated primary BAs to release free primary BAs and amino acids (taurine or glycine) and subsequently modify the free primary BAs to produce a broad range of secondary BAs (40). To assess the impact of HL on BA metabolism, we identified 12 metabolites in the gut that were involved in microbial BA metabolism (**Figure 4D**; **Supplementary Figures S8C, D**). For the conjugated primary BAs, the levels of GCA and GCDCA were similar between the DM group and DM-HL group, while the HL treatment resulted in significantly higher levels of TCA and TCDCA compared to the DM group. As a result of microbial deconjugation, the mice in the DM-HL group had higher levels of taurine and UDCA compared with the DM group, in combination with a significantly decreased level of CDCA. For the secondary BAs, the levels of THCA, DCA, and ACA were significantly enriched in the DM-HL group compared to the DM group. No significant difference was observed in LCA between groups, but the downstream metabolite of iso-LCA was reduced after HL treatment. These results showed that HL treatment dramatically changed the composition of the BA pool, leading to increased BA-related metabolites except for CDCA, LCA, and iso-LCA, which were the downstream metabolites of TCDCA.

To investigate the potential contribution of BA-related metabolites to the benefits of HL, we analyzed the correlations between BA-related metabolites and T2DM-related parameters using Spearman’s correlation (**Figure 4E**). We found that 11 out of 12 BA-related metabolites were significantly correlated with at least one parameter. Among these 11 metabolites, five metabolites enriched in the DM-HL group, including ACA, UDCA, TCDCA, taurine, and TCA, were negatively correlated with both body weight gain and adipocyte size. Moreover, ACA was also negatively correlated with the AUC of glucose during OGTT and insulin resistance. Two metabolites that markedly decreased in the DM-HL group, i.e., CDCA and iso-LCA, were positively correlated with body weight gain and adipocyte size. These correlations suggested that BA-related metabolites may be key mediators of the microbiota-host interaction during HL treatment, leading to the protective effect of HL on HFD/STZ-induced T2DM.

### The alteration of BAs was related to the gut microbiota

**Figure 5:**
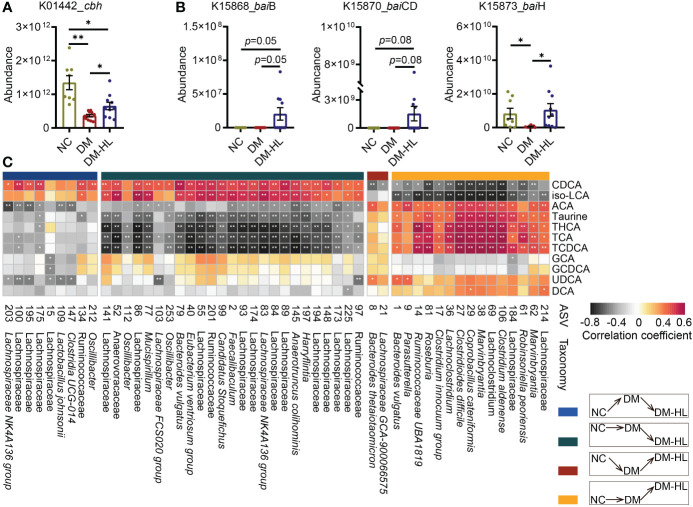
Alteration of BA composition was related to that in the gut microbiota. **(A)** The absolute abundance of the *cbh* gene predicted by PICRUSt2. **(B)** The absolute abundance of the *bai*B, *bai*CD, and *bai*H genes predicted by PICRUSt2. **(C)** The Spearman correlation between metabolites and microbes. ASVs belong to different classifications based on their inconsistent responses to HFD/STZ treatment and HL administration. The metabolites were involved in microbial bile acid metabolism in the gut. The color of the block indicates the correlation (red = positive, black = negative). The corrected *p* value was adjusted by FDR. *p < 0.05; **p < 0.01. For graph **(A, B)**, data are presented as the mean ± SEM, and significance was evaluated by an unpaired *t* test with Welch’s correction. *p < 0.05, **p < 0.01.

To further determine the role of the altered gut microbiota in mediating the composition of BAs that are potentially protective against T2DM, we investigated the absolute abundance of putative functional genes of gut microbiota involved in BA metabolism using Phylogenetic Investigation of Communities by Reconstruction of Unobserved States (PICRUSt2) analysis. We observed that the abundance of the *cbh* gene, which is responsible for the deconjugation of BAs, increased markedly following HL treatment, and the abundance of *bai* operons (*bai*B, *bai*CD, *bai*H) that encoded several essential proteins for BA dehydroxylation was also higher in the DM-HL group than in the DM group (**Figures 5A, B**). It is suggested that HL treatment might improve the capability of microbial BA metabolism. Then, Spearman’s correlation analysis was performed to identify the potential relationships between key microbes and BA-related metabolites (**Figure 5C**). In total, 11 out of 12 metabolites were correlated with at least one ASV. Among these 11 metabolites, metabolites of ACA, taurine, THCA, TCA, and TCDCA, showed a significantly positive correlation with ASVs that were enriched in the DM-HL group (mainly belonging to *Marvinbryantia*, *Clostridium*, *Coprobacillus*, *Roseburia*, *Parasutterella*, *Bacteroides*) and negative correlations with ASVs that were suppressed by HL treatment (mainly belonging to unclassified Lachnospiraceae, *Oscillibacter*). On the contrary, CDCA and iso-LCA showed the opposite trends of correlations with the gut microbiota than the above-mentioned five metabolites. Altogether, the results indicated that the enriched gut microbes in the DM-HL group might be essential for the alteration of BA composition, possibly through the improvement of microbial BA metabolism.

## Discussion

The hypoglycemic effect of HL has been well-documented (41). However, the underlying mechanisms of the effect of HL on T2DM remain to be clarified in detail. Here, we demonstrated that an ethanol extract of HL effectively prevented HFD/STZ-induced T2DM in mice. The study revealed how HL modulated gut microbiota to alter the composition of the BA pool, which might be associated with the improvement of metabolic outcomes.

We observed that several enriched chemical compounds in the DM-HL group (BBR, tyrosol, yamogenin, 26-hydroxyecdysone, 2,4-dihydroxyacetophenone) might be derived from HL extract, contributing to the efficiency of HL in treating diabetes. It is well known that BBR exerts excellent anti-inflammatory and anti-diabetic activity (19, 20). In addition, tyrosol is also well-known for its antioxidant property and was reported to decrease the glucose level, as well as promote adipose thermogenesis to inhibit obesity in mice (42, 43). Moreover, yamogenin can inhibit TG accumulation and the expression of fatty acid synthesis-related genes (*FAS*, *SREBP1c*) in HepG2 hepatocytes (44). Thus, thepharmacological potency of HL against T2DM might be related to the fact that multiple compounds regulate targets in multiple pathways, which provides a reasonable explanation as to the better hypoglycemic efficiency of HL compared to BBR.

In the current study, we showed the reduced richness and diversity of gut microbiota in the DM-HL group compared to the DM group, and most ASVs of the gut microbiota belonging to various taxa were almost eliminated by HL, which reflected the antibacterial activity of HL. A study that used HL (Huanglian Decoction) as an agent in stressed mice with ulcers reported similar results (45). Previously, HL exhibited a good therapeutic effect on bacterial infectious diseases in the clinic and exerted a broad spectrum of antibacterial activities, especially against opportunistic pathogens, such as *Pseudomonas*, *Staphylococcus*, and *Salmonella* (41, 46–48) These findings suggest that the reduced diversity of the gut microbiota after HL treatment might be associated with its broad anti-bacterial effect. However, the underlying antibacterial role of HL still needs further investigation.

The BAs (including UDCA, THCA, and DCA) generated by the gut microbiota through various pathways (deconjugation, dihydroxylation, oxidation, and epimerization) (40) increased in the DM-HL group compared to the DM group, which indicated the alteration of microbial BA metabolism. In the current study, a gut microbiota predominated by *Bacteroides* and the *Clostridium innocuum* group was constructed in our DM-HL group, with increases in genes (*cbh*, *bai*) associated with microbial BA metabolism Previously, *Bacteroides* and *Clostridium* spp. were recognized as bile salt hydrolase (BSH)-producing bacteria, contributing to BA metabolism in the gut (49–51). These findings indicated that, in our study, HL treatment altered the composition of secondary BAs, possibly by expanding the capacity of microbial BA metabolism. However, we also noticed that the downstream metabolites of TCDCA, CDCA, and iso-LCA were markedly decreased by HL treatment, which might be explained by the substrate-specific characteristics of BSH (52). Additionally, our data suggested the promotion of hepatic BA synthesis by HL. Specifically, the up-regulation of hepatic BA synthesis genes (*Cyp7a1*, *Cyp27a1*) and the enrichment of conjugated primary BAs (TCA, TCDCA) in the gut were observed in the DM-HL group (**Supplementary Figures S8A, B**). It has been reported that hepatic BA synthesis can be partially controlled by secondary BAs by regulating FXR (53–55), and the reduction of ileal FXR signal induced by BAs accelerated BA synthesis in mice (56). In addition, CDCA is the most effective ligand for FXR, while UDCA and THCA are characterized as FXR antagonists (5, 57, 58). These findings suggest that secondary BAs in the DM-HL group might promote hepatic BA synthesis by inhibiting FXR. Collectively, our findings indicate that HL treatment affects the composition of the BA pool by modulating gut microbiota.

Close associations were observed between BAs and T2DM-related parameters, suggesting that the altered BA pool in the DM-HL group affected host health. Specifically, UDCA and DCA, which were negatively correlated with obesity, were enriched in the DM-HL group. In previous studies, UDCA and DCA were reported to reduce the uptake of long-chain fatty acids in HFD-fed mice by inhibiting the liver-specific fatty acid transport protein 5 (59). UDCA has also been reported to promote hepatic lipid excretion in feces (60). These mechanisms might explain the improved lipid accumulation in the current study. Moreover, the enrichment of THCA was also observed in the DM-HL group. The reduction of THCA has been identified in the plasma of diabetic patients, and THCA can stimulate GLP-1 secretion and maintain glucose homeostasis *in vivo* (5, 61). Thereby, our findings suggest that the modified BA pool with increased protective BAs in the DM-HL group might play an important role in T2DM treatment.

In conclusion, HL extracts that are high in various active components can effectively improve impaired glucose tolerance and lipid accumulation in T2DM mice, possibly by altering the structure of the gut microbiota and BA pool. We propose that the beneficial effects of HL on T2DM, at least partly, depend on the gut microbiota. Our study highlights the key role of the HL extract in alleviating T2DM and provides a theoretical basis for the clinical study of using HL in T2DM patients.

## Data availability statement

The datasets presented in this study can be found in online repositories. The names of the repository/repositories and accession number(s) can be found below: https://www.ncbi.nlm.nih.gov/, PRJNA859559.

## Ethics statement

All animal experimental procedures were approved by the Institutional Animal Care and Use Committee (IACUC) of Shanghai Jiao Tong University (No. A2020030).

## Author contributions

BL, TD, and XW prepared the HL extract. YZ and HX conceived the original idea. DL designed the study, conducted experiments and data analysis, and wrote the manuscript. GF performed the animal trial and sample collections and revised the manuscript. YL, HP, and PL participated in the bioinformatics analysis of the gut microbiota and metabolites; CZ designed and supervised the study, and revised the manuscript. All authors contributed to the article and approved the submitted version.
