# Supplementary material for: Benefits of Huang Lian mediated by gut microbiota on HFD/STZ-induced type 2 diabetes mellitus in mice
Source: Front Endocrinol (Lausanne). 2023 Jan 18;14:1120221. doi: 10.3389/fendo.2023.1120221 (PMC9889990; doi:10.3389/fendo.2023.1120221)
Supplement: Supplementary file 2 [file DataSheet_1.docx]

Supplementary Material

#
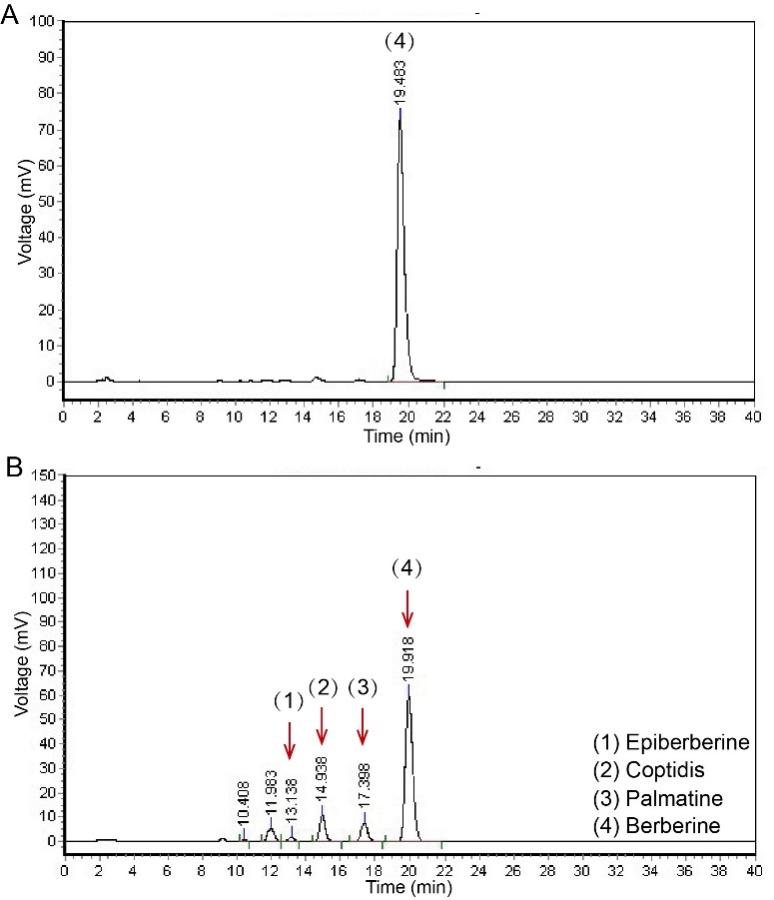
Supplementary Figures

**Supplementary Figure S1. Chemical composition of HL extract using high performance liquid chromatography (HPLC).** (A) The chromatogram of standard by HPLC. (B) The chromatogram of HL extract by HPLC.

**Supplementary Figure S2. Time course of changes in body weight gain (% 1^st^ day body weight).** Data were represented as mean ± SEM, and significance was evaluated using one-way ANOVA followed by Tukey *post-hoc* test. **p* < 0.05 *vs.* NC group; #*p* < 0.05, ##*p* <0.01, ###*p* < 0.001 *vs.* DM group.


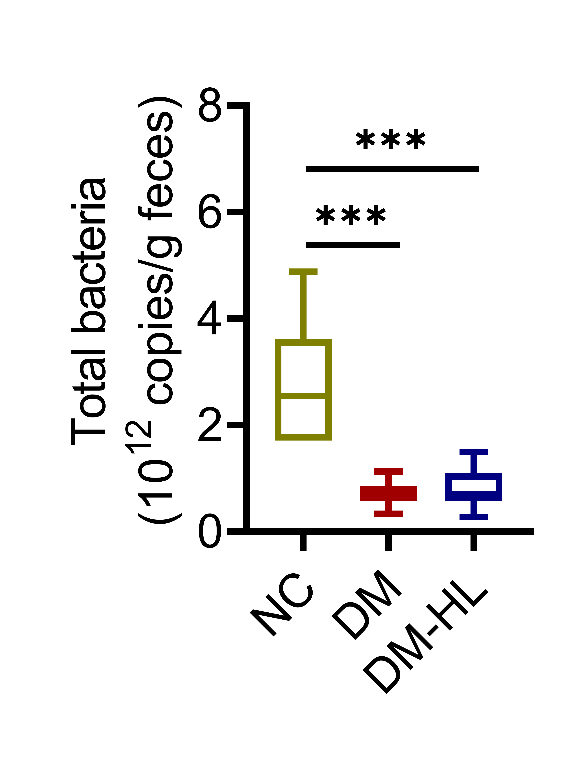


**Supplementary Figure S3. Total bacterial loads in feces among groups.** Data were shown in Boxplot. And box spans from first quartile to third quartile. Whiskers extend from maximum to minimum. Significance was evaluated using one-way ANOVA followed by Turkey post-hoc test. ****p* < 0.001.


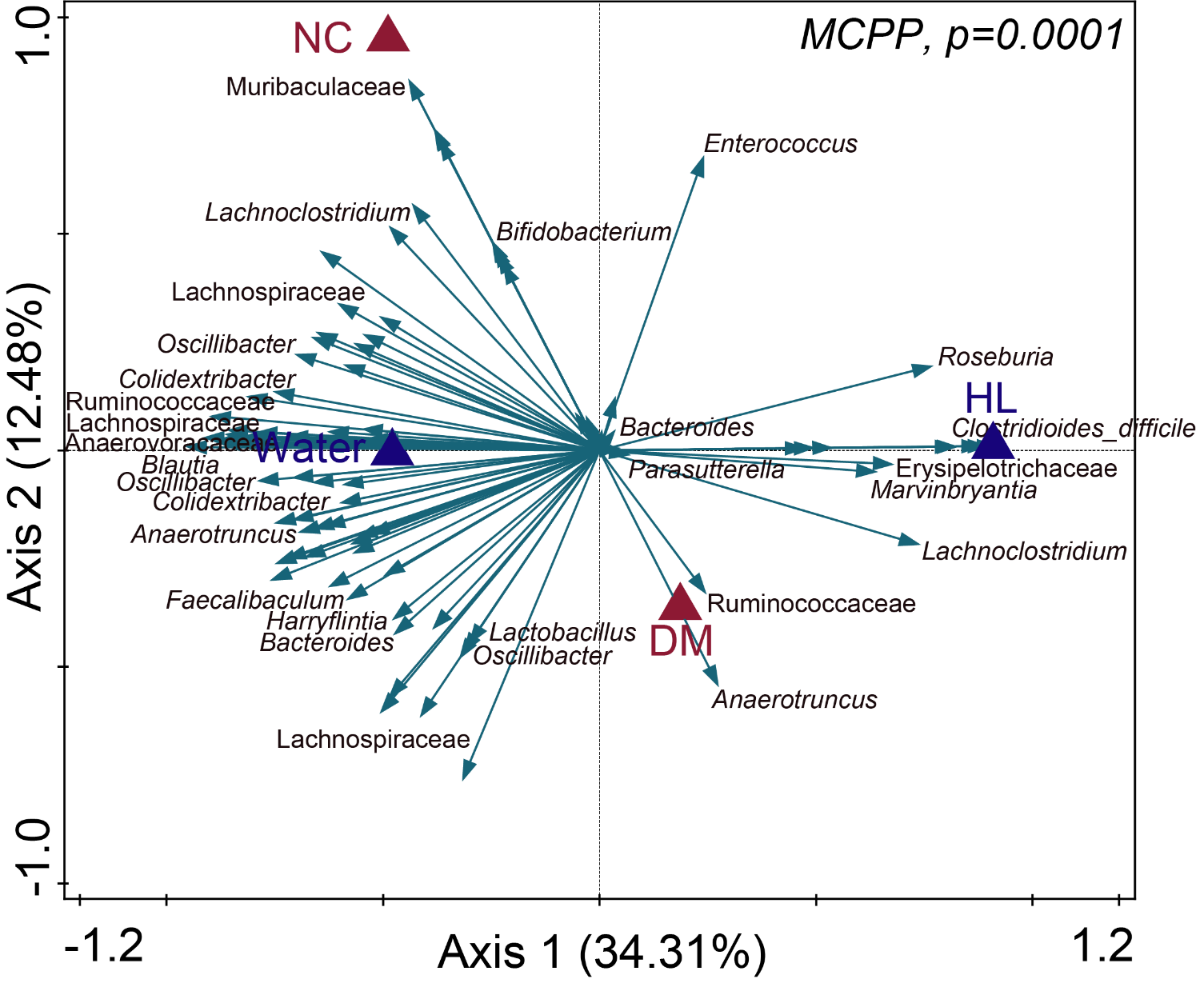


**Supplementary Figure S4. 101 ASVs were identified as key variations responding to disease model and HL administration by Redundancy analysis (RDA).** Environmental factors (Disease: NC, DM; Drug: HL and water) were represented as filled triangles. 101 ASVs that marked as blue arrows could explain at least 46.8% variations of gut microbiota among samples. The relative better-fitting variations were labeled with name at family or genus level. and the p-value was analyzed by MCPP test.

**
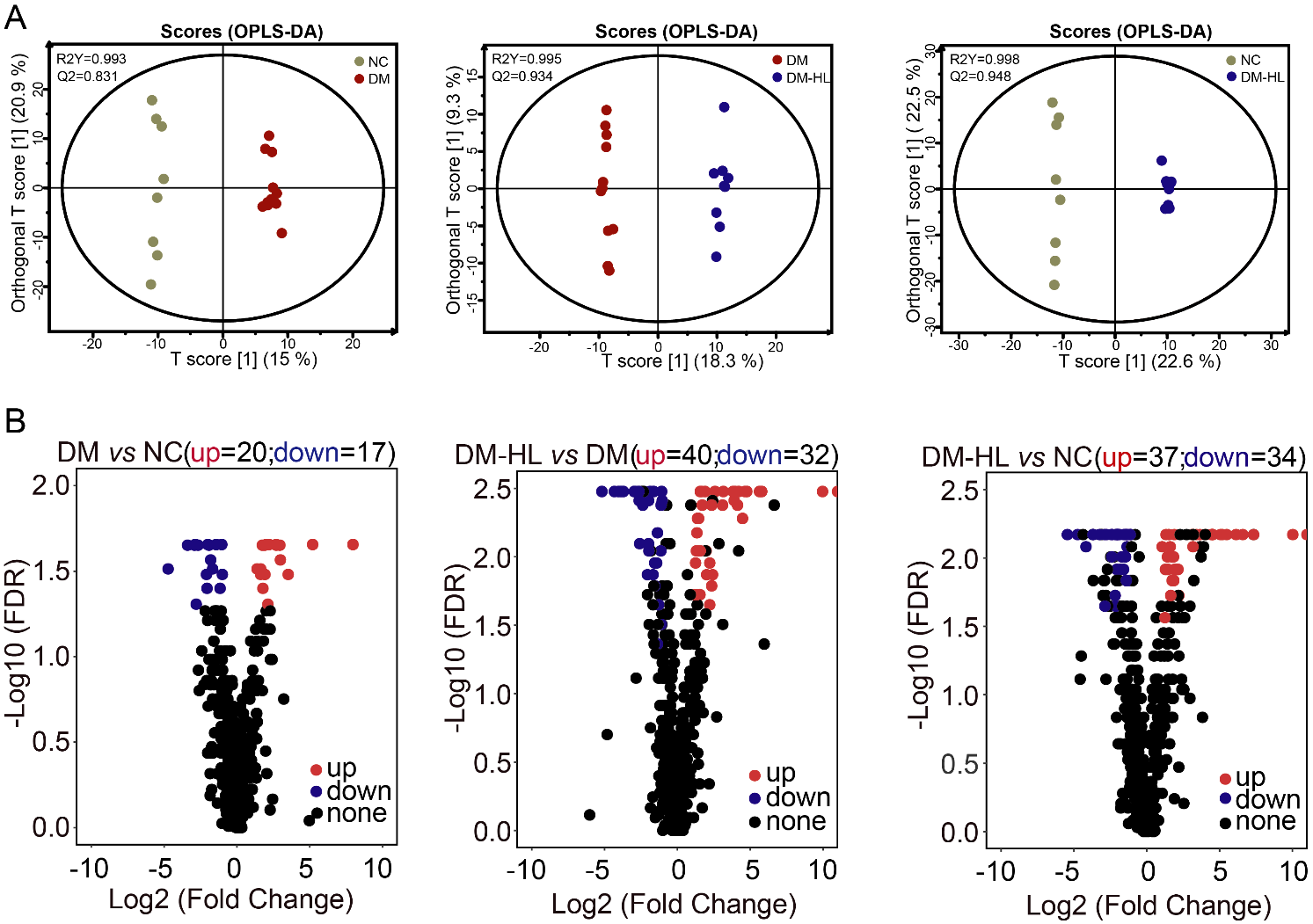
**

**Supplementary Figure S5. The alteration of** **metabolome through pairwise comparison between groups.** **(A)** The difference of overall metabolome (DM *vs.* NC; DM-HL *vs.* DM; DM-HL *vs.* NC) in the orthogonal partial least square discriminant analysis (OPLS-DA). **(B)** Volcano plot showed specific differential metabolites between groups (DM *vs.* NC; DM-HL *vs.* DM; DM-HL *vs.* NC). Red dot represented increased metabolites (FC > 2), blue dot represented reduced metabolites (FC < 0.5) with VIP score > 1.5 in OPLS-DA model, *p* < 0.05.


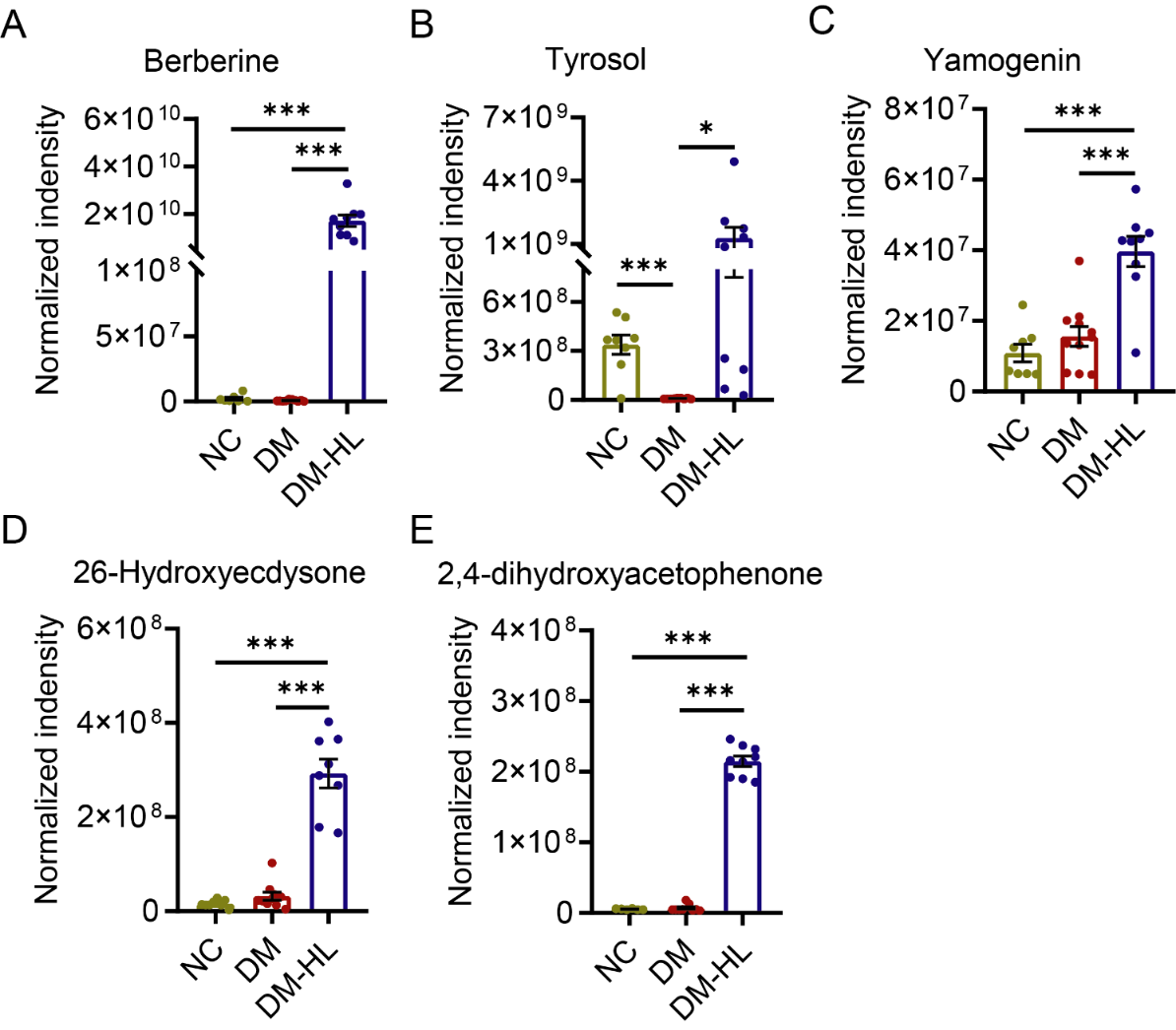
**Supplementary Figure S6. HL treatment significantly supplied herbal ingredients in colon.** **(A-E)** The relative abundance of berberine, tyrosol, yamogenin, 26-hydroxyecdysone, and 2,4-dihydroxyacetophenone, respectively. Data were represented as mean ± SEM. and significance was evaluated using Unpaired t test with Welch correction. **p* < 0.05, ****p* < 0.001.


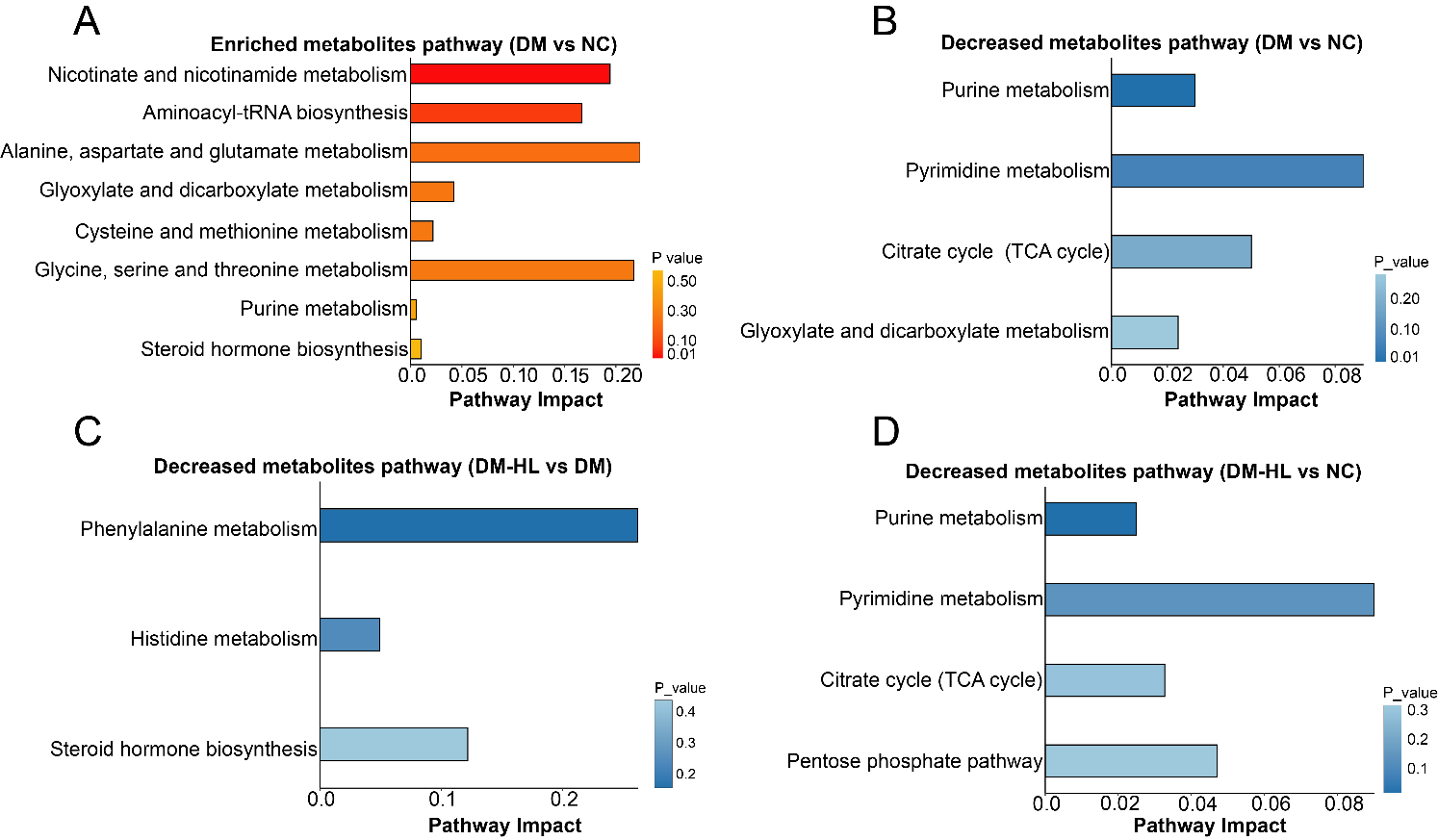
**Supplementary Figure S7. Metabolic pathway enrichment analysis through pairwise comparison between groups. (A)** Enriched metabolites pathway in DM group compared to NC group. **(B)** Decreased metabolites pathway in DM group compared to NC group. **(C)** Decreased metabolites pathway in DM-HL group compared to DM group. **(D)** Decreased metabolites pathway in DM-HL group compared to NC group.


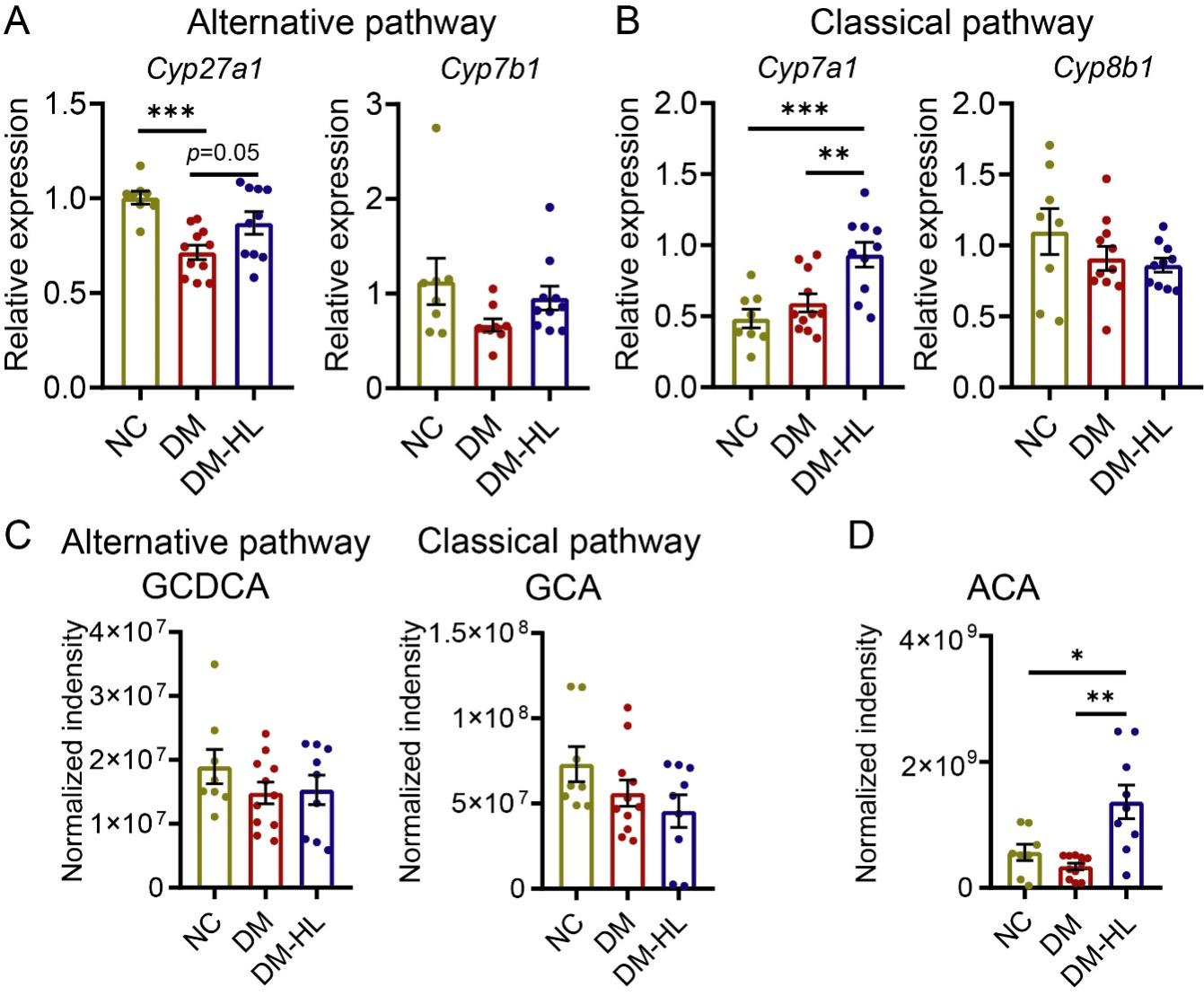


**Supplementary Figure S8. HL treatment promoted primary BAs synthesis. (A)** The expression of *Cyp27a1* and *Cyp7b1* genes, involved in alternative primary bile acids synthesis. **(B)** The expression of *Cyp7a1* and *Cyp8b1* genes, involved in classical primary bile acids synthesis. **(C)** The level of GCDCA and GCA in colon contents. **(D)** The level of ACA in colon contents. GCDCA, Glycochenodeoxycholic acid; GCA, Glycocholic acid; ACA, Allocholic acid. Data were represented as mean ± SEM. And for graph **(A, B)**, significance was evaluated by one-way ANOVA followed by Tukey *post-hoc* test. For graph **(C, D)**, significance was evaluated using Unpaired t test with Welch correction. **p* < 0.05, ***p* < 0.01, ****p* < 0.001.
